# Recombinant Human Adenovirus Type 5 Co-expressing RABV G and SFTSV Gn Induces Protective Immunity Against Rabies Virus and Severe Fever With Thrombocytopenia Syndrome Virus in Mice

**DOI:** 10.3389/fmicb.2020.01473

**Published:** 2020-06-30

**Authors:** Zhongxin Zhao, Wenwen Zheng, Lina Yan, Peilu Sun, Tong Xu, Yelei Zhu, Lele Liu, Li Tian, Hongbin He, Yurong Wei, Xuexing Zheng

**Affiliations:** ^1^Department of Virology, School of Public Health, Cheeloo College of Medicine, Shandong University, Jinan, China; ^2^Key Laboratory for Biotech-Drugs Ministry of Health, Key Laboratory for Rare & Uncommon Diseases of Shandong Province, Institute of Materia Medica, Shandong Academy of Medical Sciences, Jinan, China; ^3^Zhejiang Provincial Center for Disease Control and Prevention, Hangzhou, China; ^4^Department of Biological Sciences, College of Life Sciences, Shandong Normal University, Jinan, China; ^5^Institute of Veterinary Medicine, Xinjiang Academy of Animal Science, Urumqi, China

**Keywords:** severe fever with thrombocytopenia syndrome virus, rabies virus, adenovirus type 5, bivalent vaccine, virus-neutralizing antibody

## Abstract

Both severe fever with thrombocytopenia syndrome (SFTS) and rabies are severe zoonotic diseases. As co-hosts of rabies virus (RABV) and SFTS virus (SFTSV), dogs and cats could not only be infected but also transmit the virus to human. Hence, developing a bivalent vaccine against both SFTS and rabies is urgently needed. In this study, we generated a recombinant replication-deficient human adenovirus type 5 (Ad5) co-expressing RABV G and SFTSV Gn (Ad5-G-Gn) and evaluated its immunogenicity and efficacy in mice. Ad5-G-Gn immunization activated more dendritic cells (DCs) and B cells in lymph nodes (LNs) and induced Th1-/Th2-mediated responses in splenocytes, leading to robust production of neutralizing antibodies against SFTSV and RABV. In addition, single dose of Ad5-G-Gn conferred mice complete protection against lethal RABV challenge and significantly reduced splenic SFTS viral load. Therefore, our data support further development of Ad5-G-Gn as a potential bivalent vaccine candidate against SFTS and rabies for dog and cat use.

## Introduction

Severe fever with thrombocytopenia syndrome (SFTS), first reported in China in 2009, is an emerging zoonotic disease caused by a tick-borne, negative-stranded RNA virus, termed as SFTS virus (SFTSV). SFTSV was identified in 2011 (Yu et al., 2011). Thereafter, South Korea and Japan also reported SFTS cases (Kim et al., 2013; Takahashi et al., 2014). In human cases, SFTS is characterized by fever, thrombocytopenia, and hemorrhagic tendency with mortality rates ranging from 12 to 30%. Many kinds of wild and domestic animals have been found to be susceptible to SFTSV (Hayasaka et al., 2016; Oh et al., 2016; Tabara et al., 2016; Kimura et al., 2018; Chen et al., 2019). Recently, epidemiological findings indicated that companion animals including dogs and cats are potential reservoirs of SFTSV and could support SFTSV transmission to humans (Lee et al., 2019; Matsuu et al., 2019; Kobayashi et al., 2020). Hence, cats and dogs have been given more attention and are considered as main potential infection sources of animal-to-human transmission. Therefore, developing vaccines for dog and cat use is urgent to effectively control SFTS.

SFTSV, as a member of the genus *Phlebovirus* in the family *Phenuiviridae* (Reece et al., 2018), is a spherical, enveloped virion with diameters of 80–100 nm. The genome of SFTSV consists of three segments: large (L), medium (M), and small (S). The L segment encodes the RNA-dependent RNA polymerase. The M segment encodes two envelope glycoproteins, including Gc and Gn, which play a key role in receptor binding and membrane fusion and are targets for virus-neutralizing antibodies (VNAs). The S segment encodes nucleocapsid protein (NP) and nonstructural protein (Yu et al., 2011). Previous studies have shown that Gn, one of the envelope proteins of SFTSV, is the predominant viral antigen that induces the production of VNA, which is the major effector against SFTSV (Wu et al., 2017).

Rabies is a highly lethal acute infectious disease caused by rabies virus (RABV) with the mortality rate nearly 100%. Rabies is endemic in more than 150 countries around the world and kills nearly 59,000 people every year (Ghai and Hemachudha, 2018). The number of rabies cases reported in China has consistently ranked second in the world since the late 1990’s, and 500–3,000 people die of rabies every year (Tao et al., 2019). More than 95% of human rabies cases are transmitted by dogs or cats in China (Hu et al., 2009; Song et al., 2014). Therefore, vaccination of dogs and cats is the most efficient way to control human rabies. However, the compulsory immunization of dogs and cats is hindered by the high cost of traditional inactivated rabies vaccines in China. Hence, developing an affordable and efficacious vaccine for rabies control is in urgent need.

RABV, as a member of *Lyssavirus* genus, *Rhabdoviridae* family, is enveloped. The genome of RABV is a single-stranded, non-segmented negative-sense RNA, which encodes five structural proteins: nucleoprotein (N), phosphoprotein (P), matrix protein (M), glycoprotein (G), and RNA-dependent RNA polymerase (L). G protein is the only protective antigen of RABV and can induce VNA production (Johnson et al., 2010).

Both SFTS and rabies are serious zoonotic diseases and impose severe threat to public health. Given that SFTSV and RABV share hosts and rabies vaccine is compulsory for cats and dogs in China, development of a bivalent vaccine targeting both RABV and SFTSV could be a more promising strategy for the prevention of SFTS and rabies.

Human adenovirus type 5 (Ad5) vectors have so far been successfully utilized to develop a variety of recombinant vaccines, including the Zika virus (Guo et al., 2018), dengue virus (Khanam et al., 2009), and Ebola virus (Zhu et al., 2017). The safety and immunogenicity of Ad5-based vaccines have been highlighted by *in vitro* study, animal models, and clinical trials (Zhu et al., 2017). In this study, we generated a recombinant Ad5 encoding RABV G and SFTSV Gn (Ad5-G-Gn) and confirmed its protective roles against both RABV and SFTSV infection in mice. Furthermore, recombinant Ad5-G-Gn-induced dendritic cells (DCs) recruitment and activation and B and T cells activation, enhanced VNA production in mice.

## Materials and Methods

### Cells, Viruses, Antibodies, and Animals

Baby hamster kidney cells (BHK-21), 293T cells, and 293A cells were maintained in Dulbecco’s modified Eagle’s medium (DMEM; Gibco, Grand Island, NY) supplemented with 10% heat-inactivated fetal bovine serum (FBS; Gibco, Grand Island, NY) and 1% antibiotics at 37°C with 5% CO_2_. RABV strain CVS-11, HuNPB3, and SRV9 were propagated in NA cells. SFTSV (JS-2011-013-1 strain) was propagated in Vero cells. Fluorescein isothiocyanate (FITC)-conjugated antibody against the RABV N protein was purchased from Fujirebio Diagnostics, Inc. (Malvern, PA, USA). Monoclonal antibody against SFTSV Gn was gifted by Dr. Xue-jie Yu. Monoclonal antibody against RABV G was purchased from Millipore, Inc. (Massachusetts, USA). The FITC-conjugated goat anti-mouse IgG and goat anti-mouse horseradish peroxidase (HRP) conjugated antibody were purchased from Abcam, Inc. (Cambridge, UK). Antibodies used for flow cytometry analysis, including APC-CD19, FITC-CD40, PE-CD86, PE-Cy7-CD11c, FITC-MHC I, and PE-MHC II were purchased from BD Biosciences (Franklin, USA). The G and Gn proteins used for ELISA were purchased from Ningbo MaiYue Biotechnology Co. Ltd. (Zhejiang, China).

Six-to-eight-week-old C57/BL6 male mice and BALB/c female mice were purchased from Jinan Pengyue Experimental Animal Center. The mice were kept in an environmentally controlled room with a 12 h light-12 h dark cycle and pathogen-free food and water were provided. The experimental protocols were approved by the Institutional Animal Care and Use Committee (20180322).

### Generation of Recombinant Adenovirus

**Figure 1:**
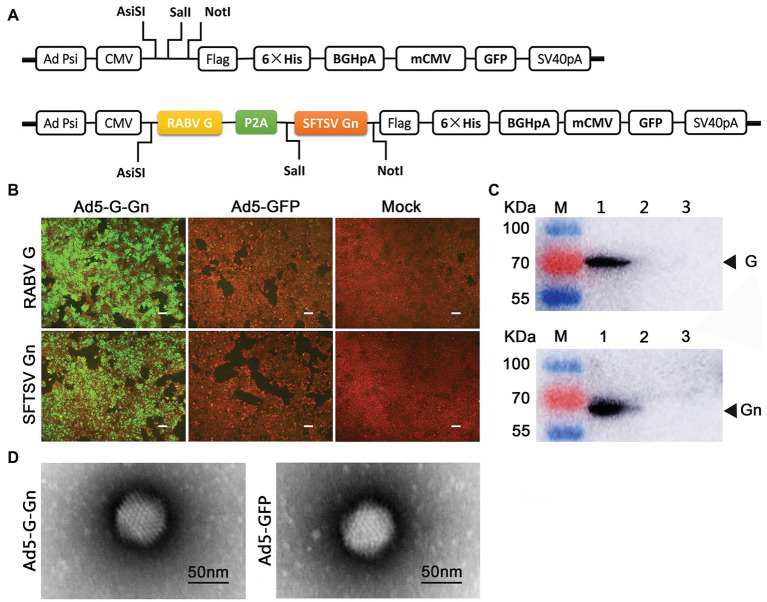
Construction and characterization of recombinant adenovirus type 5 (Ad5) expressing both RABV G and SFTSV Gn (Ad5-G-Gn). **(A)** Schematic diagram of the recombinant Ad5-G-Gn. **(B,C)** IFA **(B)** and Western blot **(C)** analysis of G/Gn expression in Ad5-G-Gn transfected 293T cells. Scale bar: 200 μm. Lane 1, Ad5-G-Gn; Lane 2, Ad5-GFP; and Lane 3, mock. **(D)** Morphology of recombinant adenovirus Ad5-G-Gn and Ad5-GFP. Scale bar: 50 nm.

The Ad5 was used as the vector to express target proteins. The construction of the recombinant Ad5-G-Gn carrying the RABV (SRV9 strain) G and SFTSV (JS-2011-013-1 strain) Gn coding sequence, linked by P2A gene, was conducted, as previously described (Zheng et al., 2015). Schematic diagram of the Ad5-G-Gn was shown in Figure 1A. After rescue and purification, the virus titer was determined in 293T cells using GFP-labeled method.

### Immunofluorescence Assay

293T cells were grown in 96-well plates and inoculated with Ad5-G-Gn or Ad5-GFP at a MOI of 1 or mock-inoculated with DMEM in the same volume. Throughout post-inoculation, cells were fixed with 80% acetone overnight at −20°C. Thereafter, cells were incubated with mouse anti-RABV-G antibody at a dilution of 1:300 or mouse anti-SFTSV-Gn antibody at a dilution of 1:400 for 1 h at 37°C and washed three times with phosphate buffer solution (PBS), and then the cells were incubated with FITC-conjugated goat-anti-mouse IgG for 1 h at 37°C, washed three times with PBS, and observed with a fluorescence microscope.

### Electron Microscopy

To identify the morphological structure of the recombinant virus Ad5-G-Gn, Ad5-G-Gn, and Ad5-GFP virions were characterized by transmission electron microscopy (TEM). The viruses were stained with 1% sodium phosphotungstate and then observed *via* TEM (Tecnai™, FEI, USA; Gai et al., 2017b).

### Western Blot

293T cells were grown in six-well-plate and infected by Ad5-G-Gn or Ad5-GFP at an MOI of 6, or mock-infected with DMEM in the same volume. At 72 h post-infection, the cells were harvested and centrifuged at 16,000 rpm for 10 min at 4°C. The supernatant was collected and separated by 10% SDS-PAGE under denaturing conditions for Western blot analyses with mouse anti-RABV-G antibody or mouse anti-SFTSV-Gn antibody. The blots were incubated with goat anti-mouse HRP conjugated antibody and developed in chemiluminescence solution. Target protein bands were observed with ultra-sensitive multi-function chemiluminescence imager (General Electric Co., USA).

### Immunization and Virus Challenge Assay

C57/BL6 male mice (6–8-weeks old) were immunized intramuscularly (i.m.) with 100 μl (10^8^ GFU) of Ad5-G-Gn, Ad5-GFP, or mock immunized with the same volume of DMEM (Sharpe et al., 2002; Hashem et al., 2019). Body weights, mental state, and dietary status were observed daily for 21 days post immunization (dpi). At 28 dpi, mice were challenged with 100 mouse lethal dose 50% (MLD_50_) of RABV HuNPB3 or 1 × 10^6^ TCID_50_ of SFTSV (JS-2011-013-1 strain) by the i.m. route. The clinical signs of RABV group were closely monitored. The mice that developed clinical signs of rabies were euthanized for humanitarian reasons. At 3 and 7 days post SFTSV inoculation, the spleen RNA was extracted with RNAprep Pure Tissue Kit [TIANGEN Biotech (Beijing) Co. Ltd., China.] for fluorescence quantitative polymerase chain reaction (PCR; DaAnGene Co. Ltd., China) to assess the SFTS viral load in the spleens following the manufacturer’s instructions.

### Assessment of Virus Neutralizing Antibodies

Blood samples were collected at 2, 4, and 8 weeks after immunization to detect titers of RABV neutralizing antibodies and SFTSV neutralizing antibodies. Titers of RABV neutralizing antibodies were confirmed by fluorescent antibody virus neutralization (FAVN) test and expressed in international units per milliliter (IU/ml; Kim et al., 2018). Titers of SFTSV neutralizing antibodies were confirmed by focus reduction neutralization test (FRNT). Hundred TCID_50_ of SFTSV JS-2011-013-1 strain were incubated with two-fold serially diluted serum from 1:8 to 1:2,048 at 37°C for 1 h, and then the mixtures of virus and serum were transferred to the 96-well plate lined with Vero cells to incubate for 2 h at 37°C. The titers of SFTSV neutralizing antibodies were calculated by Reed-Muench method.

### ELISA of RABV and SFTSV Specific Ig/IgG Antibody Titers

ELISA plates were coated with 800 ng/well RABV G protein or SFTSV Gn protein overnight at 4°C. After washing and blocking, 100 μl of 10-fold serially diluted serum was added and incubated overnight at 4°C. The plates were washed and then incubated at room temperature for 2 h with 100 μl of HRP-conjugated goat anti-mouse Ig, IgG2c, and IgG1 at a dilution of 1:500. After the plates were washed, ABTS was added and incubated at 37°C for 15 min. The absorbance at 405 nm was measured using microplate reader (Mutishan MK3, Thermo Scientific). Serum samples from mock-immunized mice were used as negative controls. A sample was deemed positive if the absorbance reading was at least 2.1 times that of the mock group. The positive antibody end-point titers are expressed as the reciprocals of the highest dilution of serum (Quan et al., 2007).

### Flow Cytometry

Immunocytes in the inguinal lymph nodes (LNs) were quantified by flow cytometry. Fifteen female BALB/c mice (6–8 weeks) per group were immunized i.m. with 10^8^ GFU of Ad5-G-Gn, Ad5-GFP, or DMEM. At 3, 6, and 9 dpi, single cell suspensions of inguinal LNs were harvested and stained with antibodies to B cell (CD19^+^ and CD40^+^) or DC cell (CD11c^+^, CD86^+^, MHC I^+^, and MHC II^+^) markers at 4°C for 30 min (Wen et al., 2011; Chen et al., 2014). Data collection was performed using a Beckman Coulter CytoFLEX S flow cytometer and data analysis was performed by CytExpert 2.3 software.

### Enzyme-Linked Immunospot Assay

Groups of female BALB/c mice (6–8 weeks, *n* = 4) were inoculated i.m. with 10^8^ GFU of Ad5-G-Gn, Ad5-GFP, or DMEM. Lymphocytes in spleens were isolated at 4 weeks post immunization and stimulated with 2 μg of purified inactive RABV or SFTSV for 20 h at 37°C and 5% CO_2_. The number of lymphocytes secreting IFN-γ and IL-4 was determined using the mouse IFN-γ/IL-4 ELISpot kit (Dakewe Biotech Co. Ltd., China) following the manufacturer’s instructions. Spot-forming cells (SFCs) were counted with an ELISpot plate reader (AID ELISPOT reader-iSpot, AID GmbH, and GER), and the background response was subtracted when calculating the number of SFCs.

### Statistical Analysis

Data were presented as the mean ± SEM and statistically analyzed using IBM SPSS Statistics 22.0 to determine significant differences *via* one-way ANOVA. ^*^p < 0.05 was considered a significant difference. ^*^p < 0.05; ^**^p < 0.01; ^***^p < 0.001.

## Results

### Generation and Characterization of Ad5-G-Gn

First, we cloned RABV-G and SFTSV-Gn into Ad5 vector to generate the recombinant Ad5-G-Gn (Figure 1A), which was then rescued in 293A cells. Determined by GFP-labeled method, the titer of Ad5-G-Gn was 6.0 × 10^10^ GFU/ml. Next, the recombinant Ad5-G-Gn was confirmed by examining RABV-G and SFTSV-Gn expression in Ad5-G-Gn infected 293T cells. IFA showed that Ad5-G-Gn-infected 293T cells exhibited strong signals of RABV-G and SFTSV-Gn protein. In contrast, Ad5-GFP‐ or mock-infected cells showed no fluorescence (Figure 1B). Western blot results showed that 70 and 61 KDa bands, corresponding to RABV-G and SFTSV-Gn, were detected in Ad5-G-Gn-infected cells but not in the Ad5-GFP or mock-infected cells (Figure 1C). These results demonstrated that both RABV G and SFTSV Gn were properly expressed in Ad5-G-Gn infected cells. To identify the morphological structure of the recombinant virus Ad5-G-Gn, recombinant Ad5-G-Gn, and Ad5-GFP virions were characterized by TEM. Similar to Ad5-GFP, Ad5-G-Gn showed regular icosahedron (Figure 1D), indicating that the expression of G and Gn did not affect the packaging of adenovirus.

### Pathogenicity and Immunogenicity of Recombinant Ad5-G-Gn in Mice

**Figure 2:**
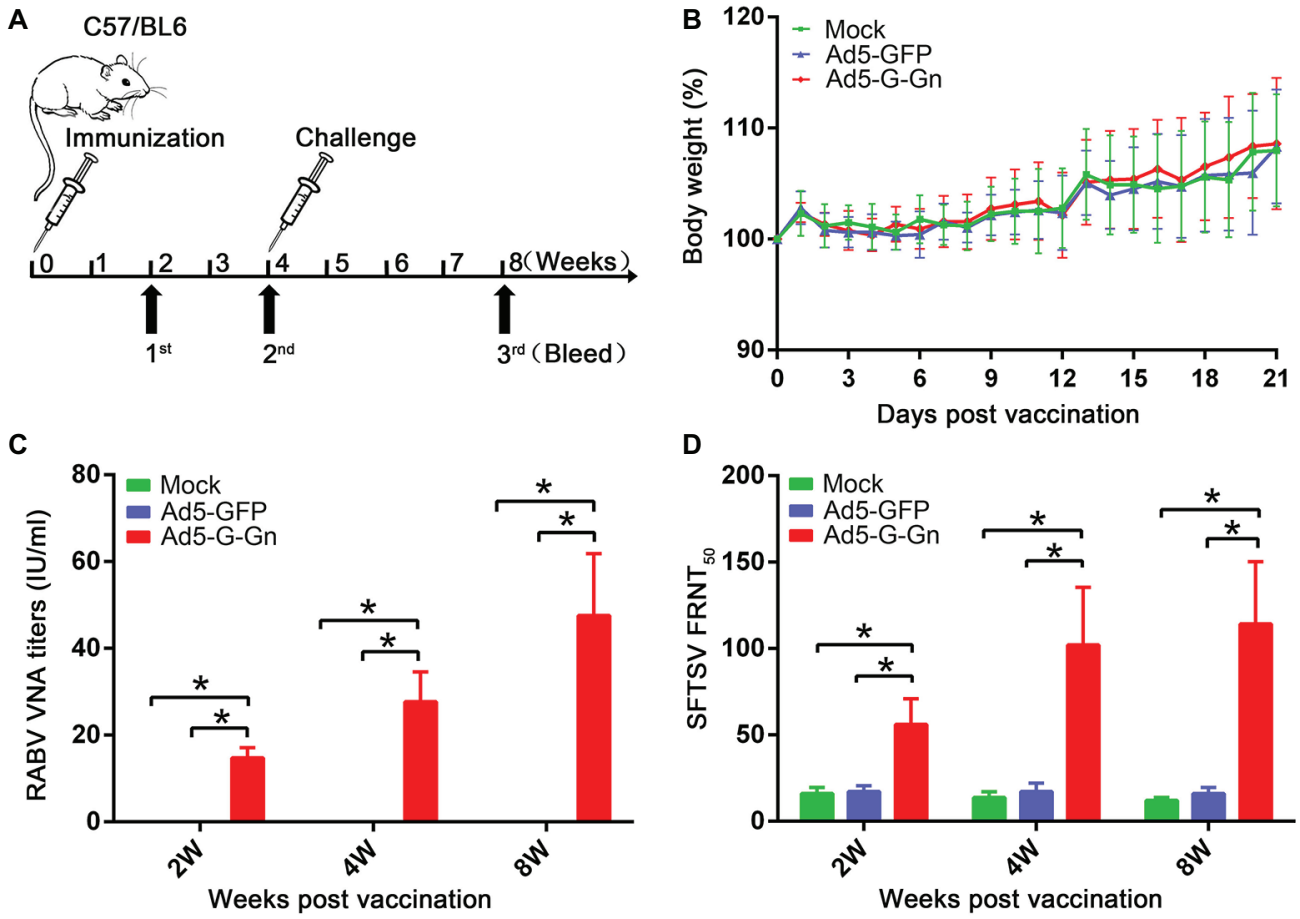
Pathogenicity and immunogenicity of recombinant Ad5-G-Gn in mice. **(A)** The procedure of immunization, serum collection, and virus challenge in mice. C57/BL6 mice (male, 6–8 weeks old) were immunized intramuscularly (i.m.) with 10^8^ GFU of Ad5-G-Gn, Ad5-GFP, or mock immunized with the same volume of Dulbecco’s modified Eagle’s medium (DMEM). Blood samples were collected at 2, 4, and 8 weeks after immunization. Viral challenge was carried out at 4 weeks after immunization. **(B)** Pathogenicity of Ad5-G-Gn in mice. Groups of C57/BL6 mice (6–8 week-old, male, *n* = 8) were immunized i.m. with 10^8^ GFU of Ad5-G-Gn, Ad5-GFP, or the same volume of DMEM (Mock), and body weights were monitored daily for 21 days. Data from all mice in each group were measured as mean values ± SEM. **(C,D)**. Groups of C57/BL6 mice (*n* = 24) were immunized with 10^8^ GFU of Ad5-G-Gn, Ad5-GFP, or mock immunized with the same volume of DMEM by the i.m. route. At 2, 4, and 8 weeks post immunization, and blood samples were collected for virus-neutralizing antibody (VNA) test. Titers of rabies virus (RABV) neutralizing antibodies were examined by fluorescent antibody virus neutralization (FAVN) test **(C)**. Titers of severe fever with thrombocytopenia syndrome virus (SFTSV) neutralizing antibodies were quantified for neutralization of SFTSV JS-2011-013-1 strain using an focus reduction neutralization test (FRNT) assay **(D)** (^*^p < 0.05).

To investigate the protective effects of recombinant Ad5-G-Gn against RABV and SFTSV, we immunized C57/BL6 mice with Ad5-G-Gn. The immune procedure of mice was shown in Figure 2A. First, body weights and growth status were recorded daily for 21 days after immunization to evaluate the pathogenicity of Ad5-G-Gn. All mice appeared normal and did not develop clinical signs (data not shown). In addition, no significant difference in body weight was found among mice inoculated with Ad5-G-Gn, Ad5-GFP, or DMEM (Figure 2B), indicating that the Ad5 vector encoding RABV G and SFTSV Gn did not affect the growth of the mice. To investigate the immunogenicity of the bivalent vaccine, sera from mice were collected at different time points after vaccination to measure VNA production. RABV VNA was examined by FAVN assay, which was recommended by Office International Des Epizooties (OIE). As shown in Figure 2C, the RABV VNA mean titers in Ad5-G-Gn immunized mice were 14.77, 27.72, and 47.57 IU/ml at 2, 4, and 8 weeks after immunization, respectively. However, no signal was detected in Ad5-GFP or mock immunized group (FAVN has a lower detection threshold of 0.02 IU/ml). As for the detection of SFTSV neutralizing antibodies, FRNT method was used. As presented in Figure 2D, the mean titers of SFTSV neutralizing antibodies in Ad5-G-Gn immunized mice were 1:56, 1:102, and 1:114 at 2, 4, and 8 weeks post immunization, respectively, which were significantly higher than that of Ad5-GFP or mock immunized group. The results suggested that the recombinant Ad5-G-Gn enhanced the production of neutralizing antibodies against both RABV and SFTSV.

### The Protective Efficacy of Ad5-G-Gn in RABV and SFTSV Infection

**Figure 3:**
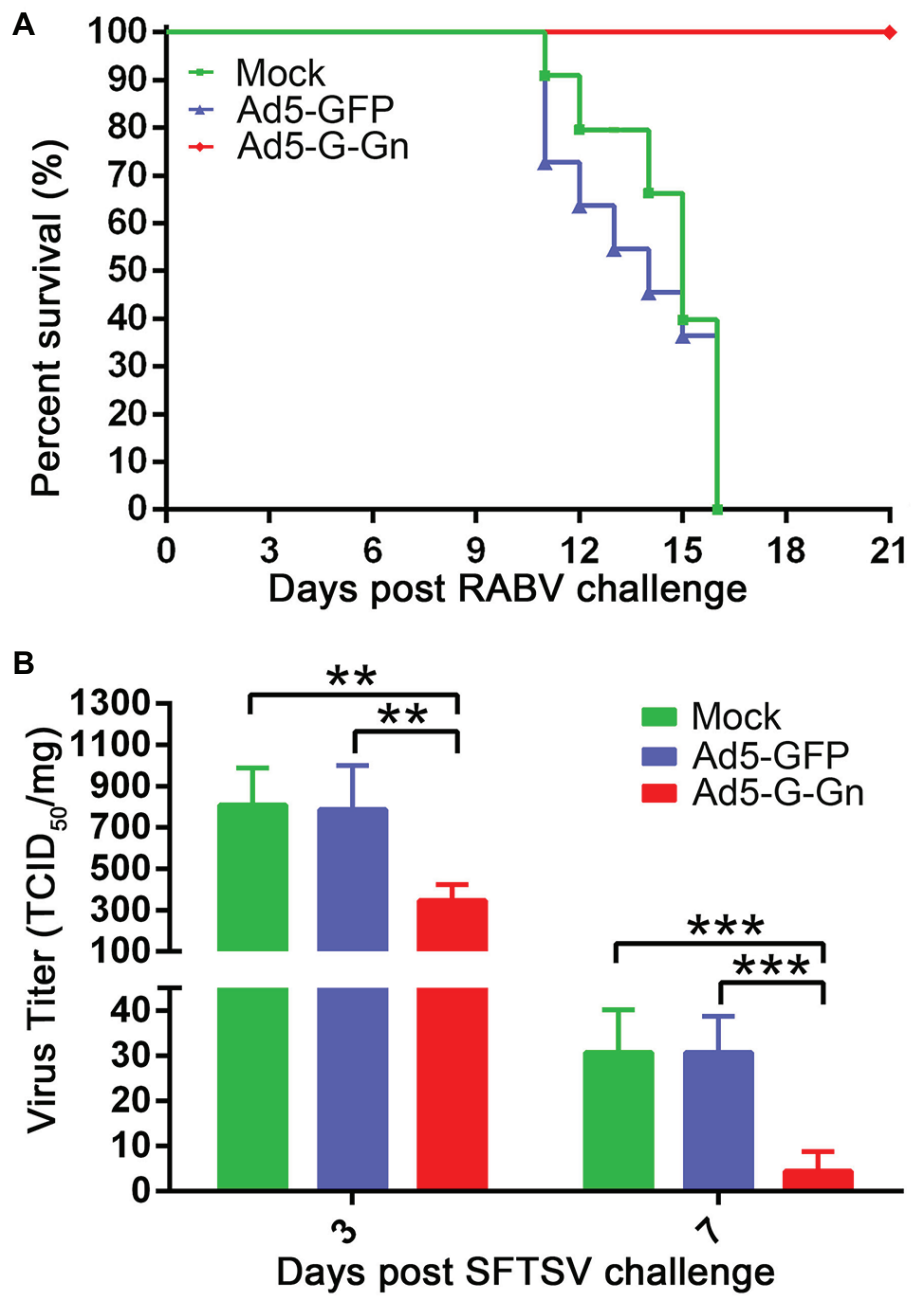
The protective roles of Ad5-G-Gn in RABV and SFTSV infection. Groups of C57/BL6 mice (*n* = 18) were immunized with 10^8^ GFU of Ad5-G-Gn, Ad5-GFP, or mocked by the i.m. route. At 28 dpi, viral challenge tests were carried out. **(A)** Survival of mice challenged with lethal RABV. At 28 dpi, mice (*n* = 8) were challenged with 100 MLD_50_ of HuNPB3 by the i.m. route and survival of mice were observed for 21 days. **(B)** SFTS viral load in spleens. At 28 dpi, mice (*n* = 10) were challenged with 1 × 10^6^ TCID_50_ of SFTSV (JS2011-013-1) by the i.m. route. SFTS viral load in spleens was detected at 3 and 7 days after viral challenge (^**^p < 0.01; ^***^p < 0.001).

Next, we investigated the protective roles of Ad5-G-Gn under the circumstances of RABV and SFTSV infection *in vivo*. Mice were challenged with 100 MLD_50_ of RABV (HuNPB3 strain). The clinical signs were closely monitored. The mice that developed general paralysis, a sign of terminal stage of rabies, were euthanized for humanitarian reasons. Ad5-G-Gn immunization provided complete protection from clinical signs of rabies following challenge with a lethal dose of RABV. In contrast, Ad5-GFP or mock group developed clinical illness and died following RABV challenge (Figure 3A). Next, we investigated the effects of Ad5-G-Gn immunization on the viral loads in SFTSV infected mice. SFTS viral loads in the spleens of mice were detected by fluorescence quantitative PCR at 3 and 7 days after SFTS viral challenge. As shown in Figure 3B, SFTS viral loads in the spleens of mice vaccinated with Ad5-G-Gn were significantly lower than that of Ad5-GFP treated or mock mice. Collectively, these results show that Ad5-G-Gn protects mice from the lethal challenge of RABV and reduces the SFTS viral load, suggesting its protective roles in RABV and SFTSV infection.

### Ad5-G-Gn Promotes the Activation of DCs, B and T Cells, and Enhances the Production of Ig

**Figure 4:**
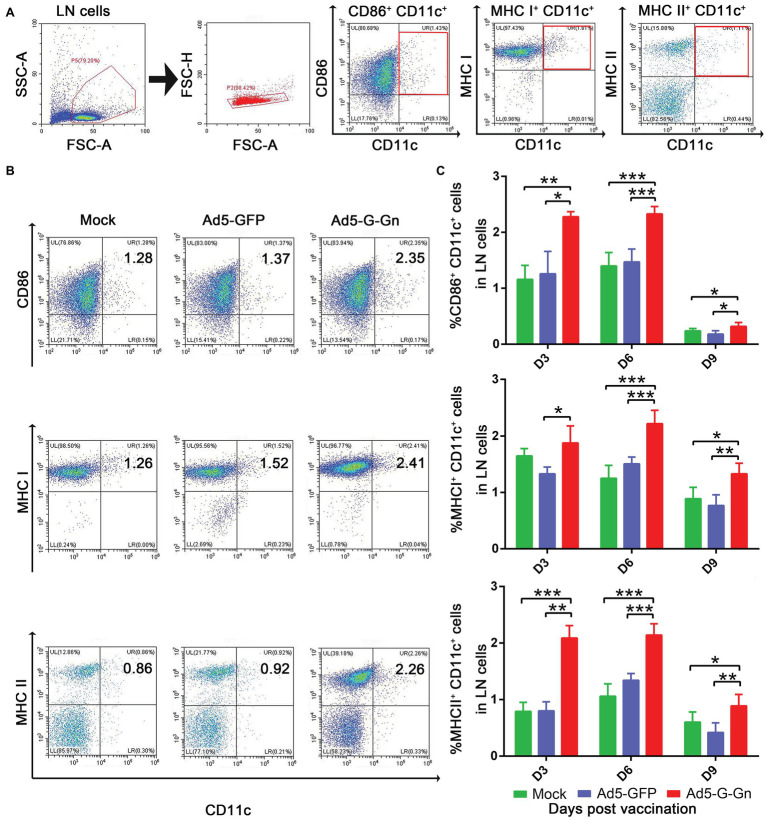
Ad5-G-Gn immunization promotes the recruitment and/or activation of dendritic cells (DCs). Groups of BALB/c mice (6–8 weeks old, female, *n* = 15) were immunized i.m. with 10^8^ GFU of Ad5-G-Gn, Ad5-GFP, or DMEM (Mock). At 3, 6, and 9 dpi, single cell suspensions of inguinal lymph nodes (LNs) were prepared and stained with antibodies against DCs activation markers and analyzed *via* flow cytometry. The gating strategies for analyzing DCs **(A)** and a representative flow cytometric plot for measuring activated DCs **(B)**. Analyses for activated DCs **(C)** (CD11c^+^ CD86^+^, CD11c^+^ MHCI^+^, and CD11c^+^ MHCII^+^) from the draining LNs at 3, 6, and 9 dpi are presented (^*^p < 0.05; ^**^p < 0.01; ^***^p < 0.001).

To investigate the functions of Ad5-G-Gn on adaptive immune responses, we examined the recruitment and activation of DCs, the activation of B and T cells, and the production of Ig. To test the ability of Ad5-G-Gn recruiting and/or activating DCs *in vivo*, activated DC cells (CD11c^+^ CD86^+^, CD11c^+^ MHCI^+^, and CD11c^+^ MHCII^+^) in the inguinal LNs were quantified by flow cytometry (Figure 4A). More DCs (Figures 4B,C) were detected in the LNs of mice immunized with Ad5-G-Gn than that of mice immunized with Ad5-GFP or DMEM. Apparently, the number of DCs recruited and/or activated by Ad5-G-Gn reached peak at 6 dpi. These results indicate that Ad5-G-Gn promotes the recruitment and/or activation of DCs in mice.

**Figure 5:**
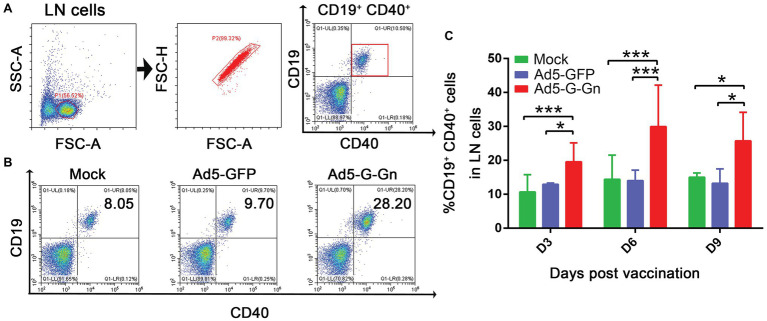
Ad5-G-Gn immunization promotes the activation of B cells. BALB/c mice were immunized with 10^8^ GFU of Ad5-G-Gn, Ad5-GFP, or DMEM (Mock) by the i.m. route. At 3, 6, and 9 days post immunization, single cell suspensions of inguinal LNs were prepared and stained with antibodies against B cell activation markers and analyzed *via* flow cytometry. The gating strategies for analyzing B cells **(A)** and a representative flow cytometric plot for measuring activated B cells **(B)**. Analyses for activated B cells **(C)** (CD19^+^ and CD40^+^) from the draining LNs at 3, 6, and 9 dpi are presented (^*^p < 0.05; ^***^p < 0.001).

**Figure 6:**
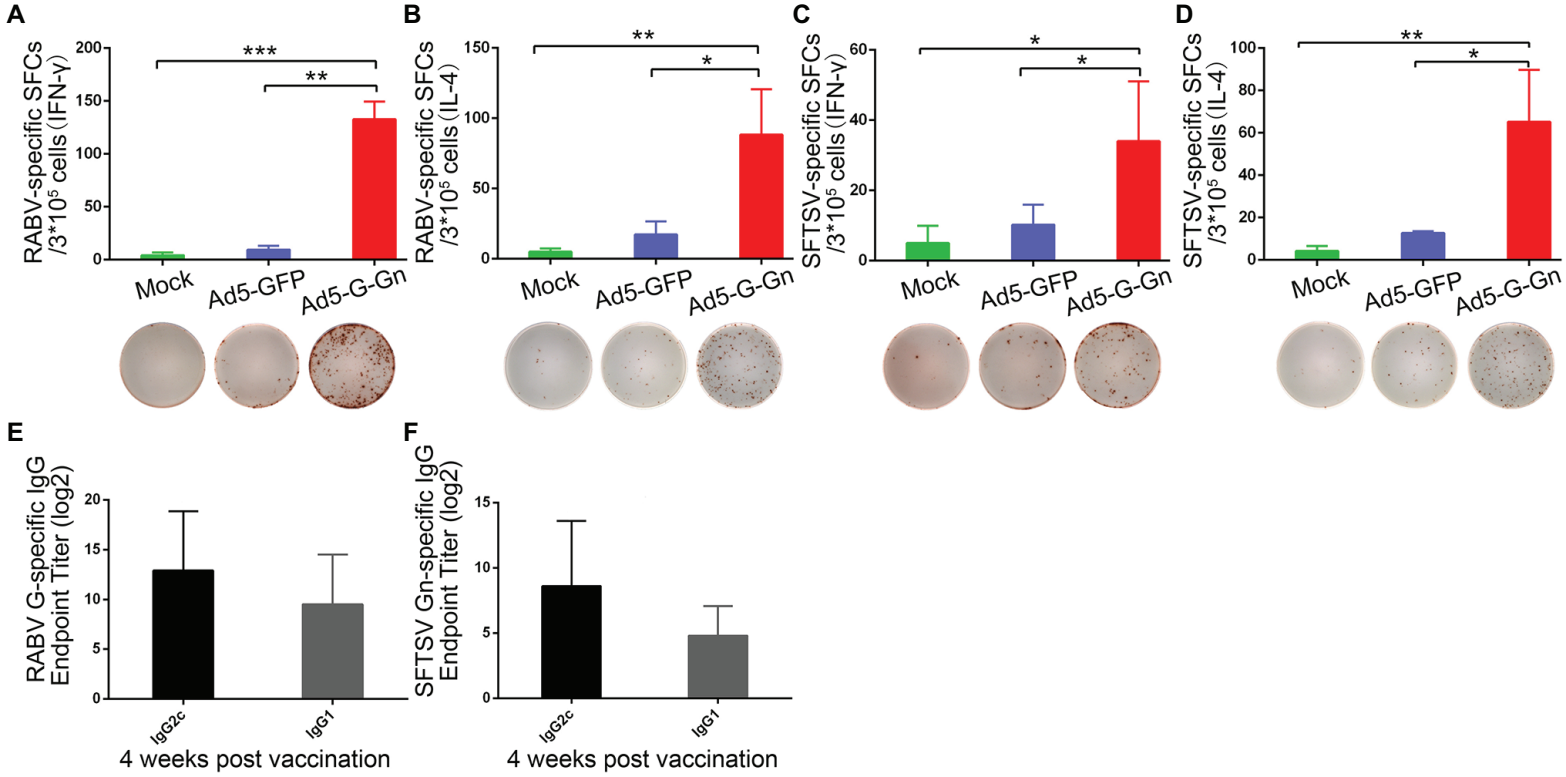
Ad5-G-Gn immunization elicits Th1-/Th2-mediated immune responses. Enzyme-linked immunospot (ELISpot) analysis of IFN-γ and IL-4 in lymphocytes from spleens of mice immunized with Ad5-G-Gn, Ad5-GFP, or DMEM (mock) at 4 weeks after immunization. **(A)** RABV specific IFN-γ spot-forming cells (SFCs). **(B)** RABV specific IL-4 SFCs. **(C)** SFTSV specific IFN-γ SFCs. **(D)** SFTSV specific IL-4 SFCs. The representative specific SFCs images were shown below each graph. IgG isotype analysis was performed at 4 weeks after immunization and serum samples were collected and subjected to ELISA analysis of IgG2c and IgG1 antibody specific to RABV G **(E)** and SFTSV Gn **(F)**. IgG titers are expressed as the reciprocals of the highest dilution of serum having a mean optical density at 405 nm greater than 2.1 times of similarly diluted negative serum samples (^*^p < 0.05; ^**^p < 0.01; ^***^p < 0.001).

Next, we examined the activation of B cells by flow cytometry. More activated B cells were detected in inguinal LNs of mice immunized with Ad5-G-Gn than that of mice immunized with Ad5-GFP or DMEM (Figure 5), indicating that immunization of Ad5-G-Gn elicited humoral immunity in mice. RABV‐ and SFTSV-specific IFN-γ and IL-4 expression in lymphocytes of spleens were detected by Enzyme-Linked Immunospot (ELISpot) assay. Ad5-G-Gn immunization greatly enhanced IFN-γ and IL-4 secretion in lymphocytes (Figure 6). Counts of RABV-specific IFN-γ and IL-4 positive cells were significantly higher in mice receiving Ad5-G-Gn than those receiving Ad5-GFP or DMEM at 4 weeks post immunization (Figures 6A,B). Similarly, the numbers of SFTSV-specific IFN-γ and IL-4 SFCs from Ad5-G-Gn group were significantly higher than those of Ad5-GFP or mock groups (Figures 6C,D). To further investigate the Th1 and Th2 mediated immune response, IgG typing analysis was performed. The titers of IgG2c and IgG1 specific to RABV-G/SFTSV-Gn were determined at 4 weeks post immunization. As presented in Figures 6E,F, increasing Th1-biased responses to RABV-G/SFTSV-Gn were elicited, which is desirable for antiviral responses (Abreu-Mota et al., 2018). These results demonstrate that single immunization of Ad5-G-Gn induces both Th1 and Th2 cell-mediated immune responses in mice with a higher Th1-biased response, contributing to the elevated VNA titers and protective efficiency.

**Figure 7:**
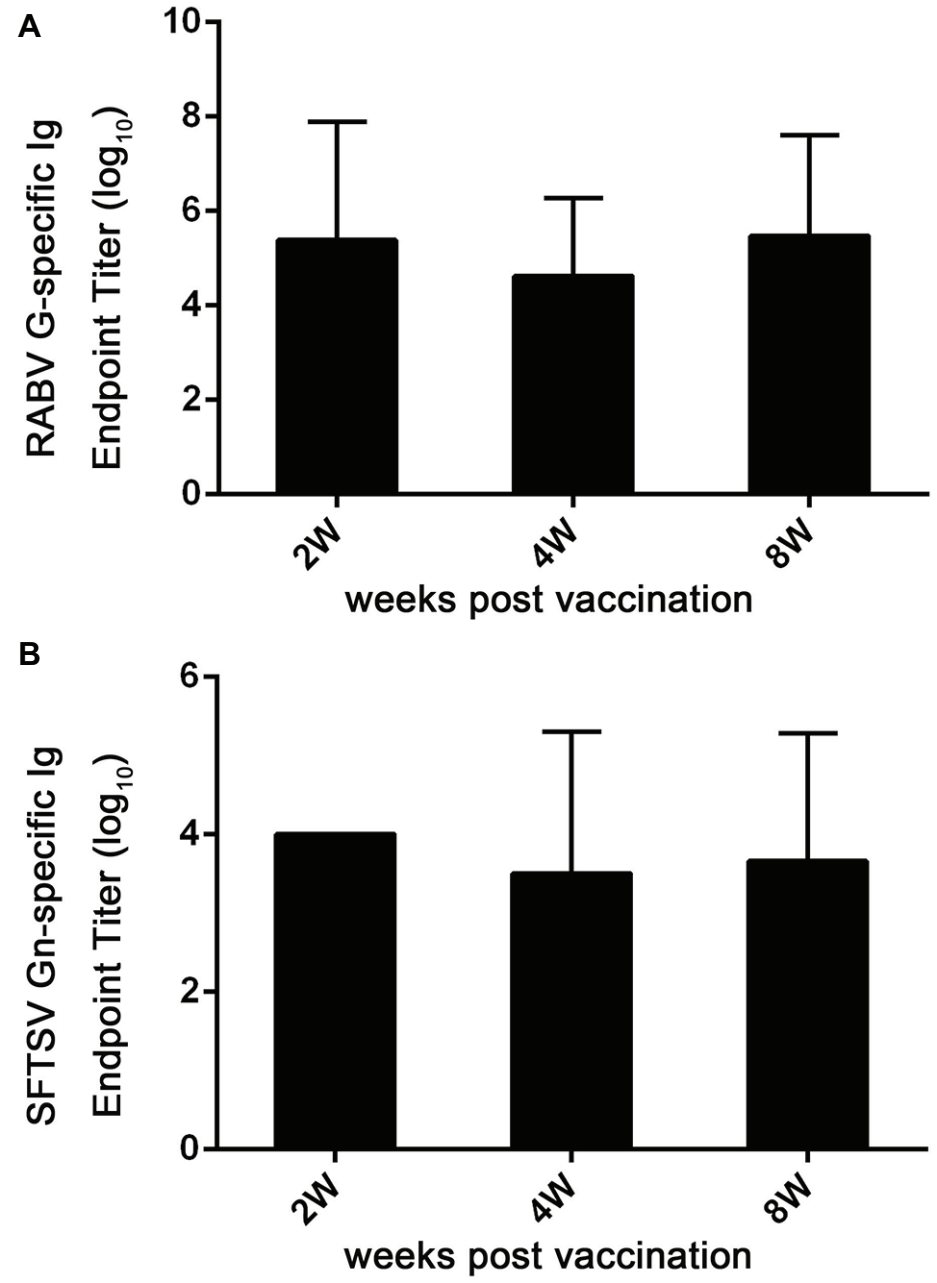
Ad5-G-Gn immunization enhances the production of Igs specific to RABV G and SFTSV Gn. At 2, 4, and 8 weeks after immunization, serum samples were collected and subjected to ELISA analysis of Ig antibodies specific to RABV G **(A)** and SFTSV Gn **(B)**. Ig titers are expressed as the reciprocals of the highest dilution of serum having a mean optical density at 405 nm greater than 2.1 times of similarly diluted negative serum samples.

To further assess immune responses induced by Ad5-G-Gn, titers of Igs against RABV and SFTSV were detected in Ad5-G-Gn immunized mice. As shown in Figure 7A, the mean titers of Ig against RABV reached 2.4 × 10^5^ at 2 weeks post immunization, and then decreased to 4.3 × 10^4^ at 4 weeks, but rapidly increased to 3 × 10^5^ at 8 weeks. The mean titers of SFTSV-specific Ig reached peak of 1 × 10^4^ at the second week, decreased to 3.3 × 10^3^ at the fourth week, and then increased to 4.6 × 10^3^ at the eighth week (Figure 7B). These data indicate that Ad5-G-Gn induce the production of Igs specific to RABV and SFTSV. Collectively, our results show that Ad5-G-Gn induces protective immunity against both RABV and SFTSV by enhancing the intensity of specific immune responses.

## Discussion

Vaccination is the most effective way to prevent rabies and SFTS. However, the high cost of traditional inactivated rabies vaccines limited their usage in developing countries. Hence, developing a novel, affordable, and long-acting rabies vaccine is urgently needed. As the major protective antigen of RABV, G protein is usually selected for novel rabies vaccine designation (Astray et al., 2017). At present, the recombinant vaccines based on poxvirus, adenovirus and parainfluenza expressing G protein of RABV have been studied, some of which have been successfully used for rabies prevention (Yarosh et al., 1996; Chen et al., 2013; Stading et al., 2017). Compared with traditional vaccines, these recombinant virus vaccines showed the advantages of better safety and immunogenicity. As for SFTS, there is no licensed vaccine for veterinary or human use. Gn and Gc, as envelope glycoproteins, containing the region for neutralizing antibodies, are the main antigen components and the hotspot of SFTS vaccine research (Wu et al., 2017). The study of DNA vaccine against SFTS indicated that Gn/Gc may be the most effective antigens to elicit protective immunity from lethal SFTSV challenge in ferrets (Kwak et al., 2019). Another study developed a live attenuated recombinant vesicular stomatitis virus (rVSV)-based vaccine candidate expressing the SFTSV Gn/Gc glycoproteins, which induced high doses of neutralizing antibodies in both immunocompetent and immune-deficient C57/BL6 mice and conferred immunodeficient mice protection from lethal SFTSV challenge (Dong et al., 2019). In the present study, a bivalent vaccine candidate against rabies and SFTS was designed based on RABV G protein and SFTSV Gn protein.

The replication-deficient Ad5 vectors, which possess good ability in inducing antibody and T cell response (Lee et al., 2017), are ideal tools for veterinary vaccine design given the low anti-Ad5 immunity in animals. The recombinant Ad5 virus expressing capsid proteins of foot-and-mouth disease virus (FMDV), as the most successful recombinant vaccine against FMDV to date, elicits effective antibody responses in cattle and provides complete protection from challenge in swine and cattle (Sreenivasa et al., 2017). José M. Rojas’s research showed that PPRV vaccine constructed using Ad5 as a vector can elicit a significant specific B and T cell responses in sheep (Rojas et al., 2014). In addition, Ad5 vectors used for vaccine development have advantages of large insert capacity and standardized bio-manufacturing process. However, the Ad5 vectors have not been utilized for a bivalent vaccine against rabies and SFTS. In the current study, we explored the potential of the Ad5 vector to express RABV G protein and SFTSV Gn protein as a bivalent vaccine.

Neutralizing antibodies play key roles in eliminating RABV, so the VNA titer is the most important index for a RABV candidate vaccine. WHO indicates that rabies VNA can achieve the group protection effect when the titer is above 0.5 IU/ml. In this study, a single dose of Ad5-G-Gn induced robust RABV-/SFTSV-specific neutralizing antibody production in mice. The RABV VNA titer reached 14.77 IU/ml at 2 weeks post Ad5-G-Gn immunization, which was far above the protective threshold defined by WHO, and the value mounted up to 47.57 IU/ml at 8 weeks post immunization, while the VNA titers of Ad5-GFP or DMEM group were under the detection threshold of FVAN test. Consistent with the high RABV neutralizing antibody level, mice immunized with Ad5-G-Gn were completely protected from lethal RABV infection, while mice of the other two control groups failed to survive the lethal challenge. Furthermore, the SFTSV VNA titer reached up to 1:114 in mice immunized with Ad5-G-Gn at 8 weeks post immunization. It has been reported that the viral RNA copies in the spleens of C57/BL6 mice were significantly higher than in the other tissues or blood after SFTSV inoculation. In addition, the detection of viral RNA sustained longer in the spleen, demonstrating SFTSV may mainly harbor in the spleen (Jin et al., 2012). Although the VNA titer threshold required for protection against SFTSV infection is unknown, we found that Ad5-G-Gn significantly reduced SFTSV viral load in spleen of mice at 3 and 7 days after SFTSV challenge.

DCs are the core of the initiation of immune response, assist to induce antigen-specific immune responses, and development of vaccines targeting DCs can increase the immunogenicity of vaccines (Hsu et al., 1996; Guzman et al., 2016). In the current study, more DCs were activated in Ad5-G-Gn immunized mice than that in Ad5-GFP or mock immunized mice. Activated DCs could present antigens to CD4^+^ T cells through MHC II, which subsequently stimulate B cells to produce antigen-specific antibodies (Cho et al., 2012). Indeed, higher VNA titers and more effective protection were observed in mice immunized with Ad5-G-Gn, which is essential for a candidate vaccine.

Also, we found that significantly more activated B cells were detected in mice immunized with Ad5-G-Gn than that in mice immunized with Ad5-GFP, suggesting a strong ability of Ad5-G-Gn to induce humoral immunity, as B cell activation is a characteristic of humoral immunity. We speculate that these differences may be caused by different exogenous proteins expressed by Ad5. G and Gn expressed by Ad5-G-Gn are the main antigenic components of RABV and SFTSV, respectively, which can induce strong immune responses. In addition, cell-mediated immune responses also play important roles in controlling viral infection. In the current study, specific IFN-γ and IL-4, one of the Th1/Th2 cytokines, respectively, secreted in lymphocytes of spleens responsive to RABV or SFTSV were highly enhanced at 4 weeks after Ad5-G-Gn immunization in mice, suggesting that both Th1 and Th2 cell-mediated immune responses were elicited by Ad5-G-Gn immunization, consistent with our previous finding that both Th1-/Th2-mediated T cell responses contribute to the elevated VNA titers (Gai et al., 2017a). Furthermore, the IgG isotype analysis showed a Th1 preferred cellular immune response, which is important for antiviral responses.

Animal models play a key role in the development of vaccines. To date, C57/BL6 mice, immunocompromised IFNAR ^−/−^ C57/BL6 mice, and aged-ferrets were used for SFTS study, among which, IFNAR ^−/−^ C57/BL6 mice and aged-ferrets are lethal SFTSV infection models that exhibit SFTS clinical features observed in human patients. However, under the condition of old age or immunocompromise, animal models cannot respond to vaccines normally and failed to truly reflect the efficacy of vaccines (Matsuno et al., 2017; Dong et al., 2019; Kwak et al., 2019). Although C57/BL6 mice did not show abnormal medical signs or significant weight loss, they mimicked the major clinical features of leukocytopenia and thrombocytopenia in SFTS patients, and the splenic virus RNA copies could be tested to evaluate the infectious status of SFTS (Jin et al., 2012). Hence, in this study, 6–8-week-old C57/BL6 mice were selected as the animal model, and splenic SFTS viral load was used as the index to evaluate the protective efficacy of vaccine candidate against SFTSV. Considering the limitations of nonlethal murine model, further safety and protective efficacy evaluations are needed.

In conclusion, we generated a recombinant Ad5-G-Gn vaccine against rabies/SFTS and evaluated its immunogenicity and efficacy in mice. Our study showed that a single immunization with Ad5-G-Gn could activate DCs and B and T cells and induce robust VNA production. Consequently, Ad5-G-Gn provided complete protection against the lethal RABV challenge and significantly reduces SFTS viral load. Thus, these data suggested Ad5-G-Gn has the potential to be exploited as a safe and efficacious bivalent vaccine against rabies and SFTS. We will further evaluate the safety and immunogenicity of Ad5-G-Gn in dogs and cats in the future study.

## Data Availability Statement

The raw data supporting the conclusions of this article will be made available by the authors, without undue reservation, to any qualified researcher.

## Ethics Statement

The animal study was strictly conducted in accordance with the recommendations of Shandong University and approved by the Institutional Animal Care and Use Committee (20180322).

## Author Contributions

XZ designed the study. ZZ, WZ, LY, PS, TX, YZ, LL, LT, HH, and YW conducted the experiments. ZZ, WZ, and XZ analyzed the data and wrote the manuscript. All authors contributed to the article and approved the submitted version.

## Conflict of Interest

The authors declare that the research was conducted in the absence of any commercial or financial relationships that could be construed as a potential conflict of interest.
